# Aggrecanase-selective tissue inhibitor of metalloproteinase-3 (TIMP3) protects articular cartilage in a surgical mouse model of osteoarthritis

**DOI:** 10.1038/s41598-020-66233-0

**Published:** 2020-06-09

**Authors:** Hiroyuki Nakamura, Phoung Vo, Ioannis Kanakis, Ke Liu, George Bou-Gharios

**Affiliations:** 1 Department of Oral and Maxillofacial Surgery, Kanazawa University Graduate School of Medical Science Kanazawa, Ishikawa, Japan; 2 Matrix Biology Department, the Kennedy Institute of Rheumatology Division, Imperial College London, Hammersmith, London, UK; 3 Institute of Ageing and Chronic Disease, University of Liverpool, William Henry Duncan Building, Liverpool, UK

**Keywords:** Drug development, Experimental models of disease, Molecular medicine, Osteoarthritis

## Abstract

A key feature of osteoarthritis is the gradual loss of articular cartilage and bone deformation, resulting in the impairment of joint function. The primary cause of cartilage destruction is considered to be the presence of elevated proteases, such as matrix metalloproteinases (MMPs) and a disintegrin and metalloproteinase with thrombospondin motifs (ADAMTSs). However, clinically tested global MMP inhibitors have low efficacy that may be due to their lack of selectivity. We previously demonstrated *in vitro* that a variant of tissue inhibitor of metalloproteinase-3 ([-1A]TIMP3) inhibits ADAMTSs but not MMPs. In this study, we tested whether the selectivity of [-1A]TIMP3 is beneficial compared with that of the wild-type TIMP3 in preventing or delaying the onset of the degenerative effects in a mouse model of osteoarthritis. We generated transgenic mice that overexpressed TIMP3 or [-1A]TIMP3 driven by a chondrocyte-specific type II collagen promoter. TIMP3 transgenic mice showed compromised bone integrity as opposed to [-1A]TIMP3 mice. After surgically induced joint instability, TIMP3 overexpression proved to be less protective in cartilage destruction than [-1A]TIMP3 at late stages of OA. The selective inhibition of ADAMTSs provides the possibility of modifying TIMP3 to specifically target a class of cartilage-degrading proteinases and to minimize adverse effects on bone and possibly other tissues.

## Introduction

Osteoarthritis (OA) is a pathological condition resulting from the degradation of articular cartilage^1^. The depletion of aggrecan is considered as an early critical event for OA progression. Aggrecan is degraded by matrix metalloproteinases (MMPs) and aggrecanases. The first aggrecanase identified was ADAMTS-4 (aggrecanase-1)^2^, and since then, ADAMTS-1^3^, ADAMTS-5 (aggrecanase-2)^4^, ADAMTS-8^5^, ADAMTS-9^6^, and ADAMTS-15^7^ have been shown to possess aggrecanolytic activity. On the other hand, collagen is degraded by members of the MMP family, including MMP-1, -2, -8, -9^8^, and -3^9^, which are found elevated in humans with rheumatoid arthritis or osteoarthritis. The levels of MMP-1, -3, -13, and -14 (MT1-MMP)^10^ and ADAMTS-4, -5^10^, and -16^11^ are higher in OA cartilage than in non-arthritic cartilage.

Although some of the developed broad-spectrum or more specific MMP inhibitors have shown impressive effects on the preclinical animal models of OA, only few have entered clinical trials for patients with mild to moderate knee OA^12^. The main problem being off-target effects, where the occurrence of tendonitis-like fibromyalgia or musculoskeletal syndrome (MSS) halted several trials. Thus, the lack of the selectivity and off-target effect of these MMP inhibitors has been proposed as a possible explanation of their ineffectiveness. In order to identify the metalloproteinases responsible for degrading the cartilage ECM in OA and could, therefore, be the best targets for therapeutic intervention, MMP- or ADAMTS-knockout mice have been generated. MMP-3-knockout mice were shown to be protected from spontaneously developing OA over time^13^; however, when OA was surgically induced, they developed similar or even more severe OA compared with wild-type controls^13^. In contrast, ADAMTS-5^14^ and MMP13^15^ knockout mice knee joints were protected from destruction in the destabilization of the medial meniscus (DMM) mouse OA model. ADAMTS-5 null knee cartilage were also protected using the antigen-induced RA model^16^. Similarly, MM9 knock out were protected from antibody induced arthritis^17^, suggesting that both these enzymes play crucial role in the development of arthritis.

In order to inhibit these enzymes, mammals synthesise tissue inhibitors of metalloproteinases (TIMPs), four of which have been identified in humans and mice and characterised as endogenous inhibitors of collagenases and aggrecanases. We have previously demonstrated that TIMP3 inhibits ADAMTS-4 and -5 with sub-nanomolar K_i_ values^18^. Moreover, the N-terminal inhibitory domain of TIMP3 (N-TIMP3) effectively blocked IL-1a-induced cartilage degradation, while TIMP-1 and TIMP-2 were not effective^19^. These results suggest that TIMP3 may be an excellent cartilage protectant against cartilage degradation *in vivo*. Furthermore, the addition of an extra alanine to the N-terminus of N-TIMP3 (N-[-1A]TIMP3) results in the loss of the inhibitory activity against MMPs; however, this function is retained in most aggrecanases^20^. The present study aimed to investigate whether inhibiting a class of enzyme: either aggrecanases or collagenases is sufficient in protecting the cartilage from the onset of OA using surgically induced OA model in mice overexpressing aggrecanase-specific inhibitor ([-1A]TIMP3) compared with a broad-spectrum TIMP3 inhibitor.

## Results

### Generation of [-1A]TIMP3 transgenic mice and transgene expression

**Figure 1:**
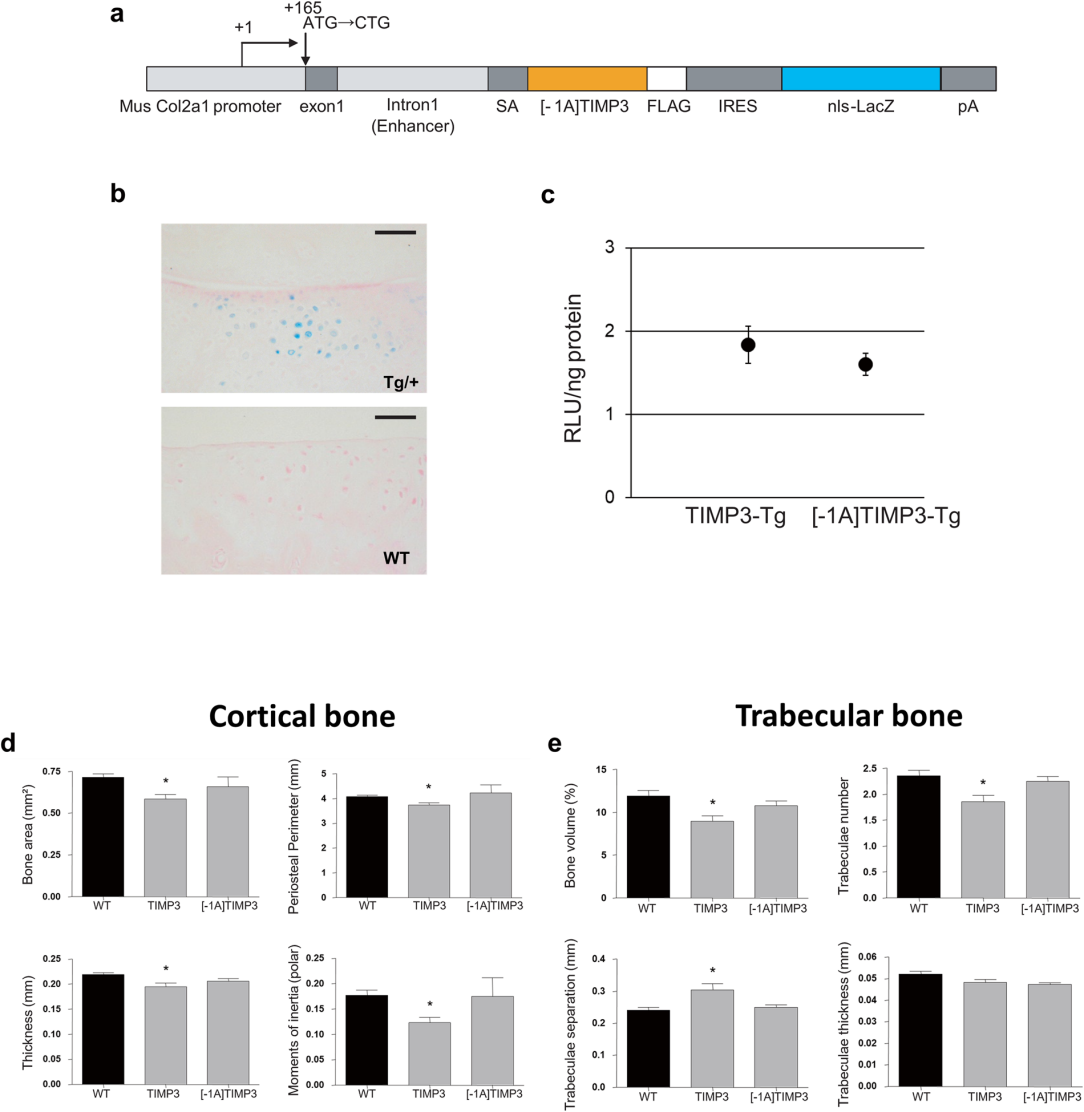
Generation of [-1A]TIMP3 transgenic mice. (**a**) Schematic representation of the construct used to generate transgenic mice. Collagen 2α1 chain (Col 2a1) proximal promoter region (3000 bp), first exon (237 bp), and first intron (3020 bp) were used to induce the expression of human [-1A]TIMP3 with a FLAG epitope tag, an IRES sequence, and LacZ with a nuclear localizing signal, followed by the bovine growth hormone gene polyadenylation signal (bpA). (**b**) X-gal staining of the knee joints of the [-1A]TIMP3 heterozygotes mice (upper panel, Tg/+) or non-transgenic wild-type mice (lower panel, WT) at 2 weeks of age. Bars, 50 μm. (**c**) Comparison of transgenic expression by determining β-galactosidase activity in TIMP3 heterozygotes (n = 5, line 19) and [-1A]TIMP3 heterozygotes (n = 7, line 7). Values represent the mean ± SEM. (**d)** Data generated from µCT scans of isolated tibia at 18 weeks of age from non-transgenic mice (WT, n = 17), TIMP3 heterozygous mice (n = 9), and [-1A]TIMP3 heterozygous mice (n = 9) for the cortical bone and (**d**) for the trabecular bone. Bars, 200 μm. (**e**) Values represent the mean ± SEM. *Indicates significance (p < 0.05) compared to wild-type mice (one-way ANOVA and Dunnett’s test).

We generated several lines of transgenic mice harbouring a bi-cistronic cassette to express either [-1A]TIMP3 or TIMP3 transgenes, with β-galactosidase as reporter gene, driven by the chondrocyte specific *Col2a1* regulatory elements (Fig. 1a). To test transgene activity, X-gal staining of 2-weeks old mouse knee joints indicated that the transgenes were seen in the articular cartilage chondrocytes of the transgenic (Tg/+) mice but not in wildtype mice (WT) (Fig. 1b). In addition, since several lines were produced, we have chosen to use one line of each of the inhibitors to run the subsequent experiments, based on the comparative level of β-galactosidase activity in the [-1A]TIMP3 heterozygote line 7, similar to that in the TIMP3 heterozygote line 19 (Fig. 1c).

To evaluate if transgenic overexpression of TIMP3 or [-1A]TIMP3 causes any changes in skeletal formation, we compared the bone morphology of TIMP3-Tg and [-1A]TIMP3-Tg heterozygotic mice at skeletally matured 18 weeks of age with WT mice using μCT. Cortical bone measurements showed a significant reduction in bone area, periosteal perimeter, thickness, and polar moments of inertia, which indicates bone strength of TIMP3-Tg mice as compared to the WT and the [-1A]TIMP3-Tg mice (Fig. 1d). Similar reductions were also observed in the trabecular bone microarchitecture of TIMP3-Tg mice, which exhibited a significant decrease of trabecular bone volume, number and thickness while trabecular separation was increased in comparison to the WT and the [-1A]TIMP3-Tg mice (Fig. 1e). On the other hand, no significant differences were observed between non-transgenic WT mice and [-1A]TIMP3-Tg heterozygotes (Fig. 1e). Importantly, since the transgene expression levels were similar in [-1A]TIMP3-Tg and TIMP3-Tg heterozygotes (Fig. 1c), these μCT results suggest that overexpression of [-1A]TIMP3 did not affect skeletal integrity, unlike TIMP3. On the other hand, histological assessment of Safranin-O stained sections at 18 weeks, showed that articular cartilage proteoglycan composition is similar between both transgenic mice ([-1A]TIMP3-Tg or TIMP3-Tg) and WT mice (Fig. S1).

### Cartilage **d**egradation of TIMP3 and [-1A]TIMP3 heterozygous mice under surgically induced mechanical stress

**Figure 2:**
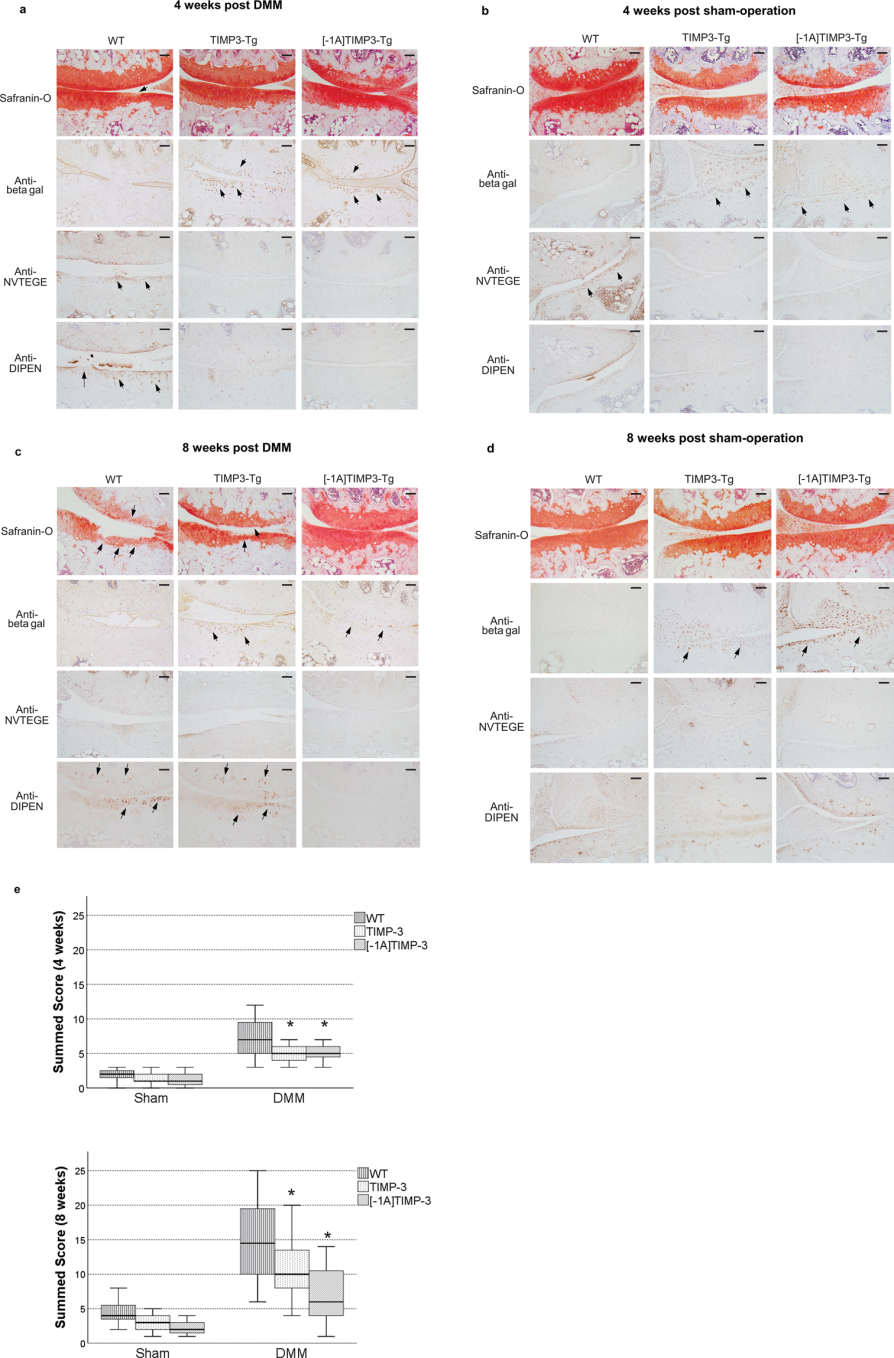
Safranin-O staining and immunostaining of sections of the medial condyle and tibial plateau of non-transgenic mice (WT), TIMP3 heterozygous mice, and [-1A]TIMP3 heterozygous mice 4 weeks after (**a**) DMM or (**b**) sham operation. β-galactosidase nuclear staining was not present in wild-type mice, but strong expression of β-galactosidase was observed in the joints of TIMP3 and [-1A]TIMP3 mice (arrows). Anti-NVTEGE and anti-DIPEN immunostaining of adjacent joint sections showed that wild-type cartilage demonstrated cleavage of aggrecan in the cartilage of the femoral condyle and tibial plateau (arrows). Bars, 50 μm. Safranin-O staining and immunostaining of sections of the medial condyle and tibial plateau of non-transgenic mice (WT), TIMP3 heterozygous mice, and [-1A]TIMP3 heterozygous mice 8 weeks after (**c**) DMM or **(d**) sham operation. No β-galactosidase staining was performed for the wild-type mice. The expression of β-galactosidase in the joints of TIMP3 and [-1A]TIMP3 mice indicate upregulated transcription of TIMP3 and [-1A]TIMP3. DIPEN immunostaining of adjacent joint sections showed that non-transgenic mice demonstrated cleavage of aggrecan in the cartilage of the tibial plateau and meniscus compared with the same areas in TIMP3 and [-1A]TIMP3 transgenic mice (arrows). Bars, 50 μm. (**e**) Box-whisker plot representing the histological scores of joints 4 and 8 weeks after the induction of joint instability or sham operation. The results are expressed as the sum of the scores from each histological section through the joints. The sample number of each group is as follows: non-transgenic mice (WT), n = 40; TIMP3, n = 15; and [-1A]TIMP3, n = 24. *Indicates significance (p < 0.05) compared to wild-type mice (one-way ANOVA and Dunnett’s test).

The next set of experiments aimed to evaluate whether the overexpression of either transgenes, TIMP3 and [-1A]TIMP3, could ameliorate OA progression in the DMM mouse model. We investigated this at 4 and 8 weeks after DMM. Four weeks after surgery, Safranin-O staining showed limited damage in non-transgenic WT mice, with weak aggrecan depletion around the loaded region (Fig. 2a). At this time point transgenic overexpression of TIMP3 or [-1A]TIMP3, verified by strong β-galactosidase immunostaining which indicated the upregulated transcription of either inhibitors, showed no remarkable changes in cartilage when compared with non-transgenic WT mice subjected to DMM (Fig. 2a). However, immunostaining using anti-NVTEGE and anti-DIPEN antibodies revealed detectable neoepitopes of aggrecan degradation at a widespread area in the non-transgenic WT mouse cartilage but not in the TIMP3-Tg or [-1A]TIMP3-Tg mice knee cartilage (Fig. 2a). Based on these observations, the knee joints at 4 weeks after surgery reflected the early stages of osteoarthritis. Thus, TIMP3 or [-1A]TIMP3 overexpression can protect the cartilage from degradation at the early stages of osteoarthritis. Sham operation showed limited NVTEGE in cartilage of the WT mice, as previously indicated in mice^21^ and human^22^ but not in either Tg mice (Fig. 2b).

At 8 weeks post DMM, the Safranin-O stained sections of knee joints showed considerable cartilage damage in non-transgenic WT mice, with loss of surface lamina and fibrillations extending down to calcified cartilage and, in some cases, erosion down to the sub-chondral bone indicating progressive OA severity (Fig. 2c). TIMP3-Tg mice also showed aggrecan loss at the superficial layer as well as further loss of surface lamina and fibrillation of the adjacent area. However, preservation of intact cartilage with no signs of degradation was seen in [-1A]TIMP3-Tg mouse knee joints, suggesting that overexpression of this inhibitor has a protective role in DMM-induced OA (Fig. 2c). Sham operation did not result in any remarkable cartilage damage in any of the transgenic or the WT mice (Fig. 2d).

All treated and sham-operated knee joints were scored at 4 and 8 weeks after surgery using OARSI recommended analysis and presented in (Fig. 2e). The data showed significant cartilage protection in either TIMP3 or [-1A]TIMP3-overexpressing mice compared with the non-transgenic WT mice following DMM, which exhibit increased OARSI scores due to OA progression Scores from sham-operated mice were low throughout the experimental period. (Fig. 2e).

To evaluate if the level of expression and therefore the concentration of [-1A]TIMP3 is important for articular cartilage protection, transgenic line 13 that showed lower transgene activity than line 7 (Fig. S2a) was subjected to DMM and showed no protection of articular cartilage at 8 weeks following DMM in Safranin-O staining (Fig. S2b), reflected in total OARSI score (Fig. S2c), indicating that the level of inhibitor in articular cartilage is critical for combatting erosion.

## Discussion

Clinical trials using inhibitors of metalloproteinases in OA, with wide inhibitory activity such as TIMPs, have highlighted the off-target effects on the musculoskeletal system which emerged for a more specific targeted therapeutic approach. Quantitative immunochemical assays by Robin Poole and colleagues in the early 1990’s revealed that aggrecan in OA undergoes two phases of pathological changes that occur in the articular cartilage; an early predominantly degenerative phase I, followed by a net reparative phase II^23^. However, despite extensive replacement of degraded proteoglycan in phase II, there is a net loss of these molecules due to OA development and progression as cartilage collagen fibres are exposed to collagenases.

Therefore, one of the current main focuses of OA pathogenesis is to provide specific inhibition of different classes of enzymes in order to evaluate the role of aggrecanases versus collagenases inhibition in OA onset and progression leading to joint function impairment. In this study, we tested whether the inhibition of aggrecanases is more effective in articular cartilage protection than using a broad-spectrum inhibitor of both collagenases and aggrecanases. For this purpose, we utilized a variant of TIMP3, [-1A]TIMP3, that provides selective inhibition of ADAMTS-4 and -5 as well as ADAM-17 (TACE)^18^ and TIMP3 which inhibits both aggrecanases and collagenases but also other adamalysins, including ADAM-10^24^, -12S^25^, and -17 (TACE)^26^. Although it is not fully understood how this selectivity is achieved with the addition of an extra alanine at the N-terminal of the molecule, the structural model suggests that TIMP3 fits tightly in the active sites of MMPs, and the addition of alanine at the N-terminal results in a different conformation of the inhibitor in the active site to accommodate the extra residue. It is assumed that the addition of alanine tilts [-1A]TIMP3 backward relative to TIMP3. Thus, in ADAMTS-4 and -5, sufficient contact sites remain with the protease to allow binding, which is not the case with collagenases. On the other hand, the inhibitory activity of [-1A]TIMP3 toward ADAMTS-4 is slightly decreased^20^. This reduction is not observed toward ADAMTS-5. These data suggested that the N-terminal region of TIMP3 is involved in inhibiting ADAMTS-4, whereas the N-terminal region is not necessary to inhibit ADAMTS-5. Eventually, [-1A]TIMP3 was a relative specific inhibitor of ADAMTS-5.

We generated transgenic mice overexpressing either [-1A]TIMP3 or TIMP3 specifically in cartilage using the chondrocyte-specific promoter/enhancer of *Col2a1* gene. While the histological findings of articular cartilage in mature [-1A]TIMP3-Tg mice were similar to that of TIMP3-Tg mice or non-transgenic WT mice, the bone structures of TIMP3-Tg mice showed significant morphological changes in the cortical compartment and trabecular network compared with [-1A]TIMP3-Tg mice which were similar to non-transgenic WT mice. These results are likely because TIMP3 overexpression represents a system with inhibition of a broad spectrum of MMPs that can affect bone homeostasis. Therefore, despite using transgenic mice with similar expression levels of [-1A]TIMP3 and TIMP3, the inhibition of MMPs appear to impair bone function, as we have shown before^27^, and evident in skeletal abnormalities demonstrated in MMP-13^28^ and MMP-14-knockout mice^29^. Similarly, MMP-2- and MMP-3-knockout mice are manifested with more severe arthritis than the wild-type mice^17^. Furthermore, MMP-14 deficiency causes arthritis due to the ablation of collagenolytic activity, which is essential for the modeling of skeletal connective tissues. Of note, TIMP3 inhibits members of the ADAM family of enzymes that control a range of cell signaling pathways. Whether ADAMs play a role in skeletal formation is currently unknown. Therefore, the possible effects of TIMP3 overexpression on the activity of ADAMs cannot be ruled out in these experiments.

To test whether this acquired selectivity of [-1A]TIMP3 is beneficial in preventing or delaying the onset of the degenerative effect in OA, the transgenic mice were subjected to surgically induced OA using the DMM mouse model. The overall results showed that overexpression of either TIMP3 or [-1A]TIMP3 can protect knee articular cartilage in the early stages of OA. The evidence for this cartilage protection is provided by the aggrecan NVTEGE and DIPEN neoepitopes, resulted from ADAMTS and MMP proteolytic activity, respectively. This revealed that early aggrecan degradation was already widespread in the cartilage of non-transgenic WT mice at 4 weeks following DMM, unlike either of the transgenic mice that were relatively protected. Safranin-O staining suggested that, although at least partly digested, aggrecan is still attached to the cartilage matrix and was hence stained by Safranin-O. This was backed up by aggrecan neoepitope immunostaining using anti-NVTEGE and anti-DIPEN compared with WT articular cartilage. This protection effect continued until 8 weeks after DMM in [-1A]TIMP3-Tg mice, where cartilage surfaces remained intact compared to WT. However, TIMP3 overexpression seemed to be less protective than [-1A]TIMP3 at the 8 weeks after DMM. Although this omni-inhibitor can inhibit both MMPs and aggrecanases to protect cartilage, TIMP3-Tg mice showed partial protection against cartilage damage, with OA scores closer, although significantly reduced, to that observed in non-transgenic DMM joints of WT mice when compared to [-1A]TIMP3-Tg mice at 8 weeks. Perhaps inhibiting a wide range of MMPs in adult mice might be detrimental for the maintenance of healthy cartilage. Inhibitors with broad activity against several MMPs, including MMP-8, -13, and -14, abrogated cartilage erosion in similar animal models of OA^30,31^. Importantly, Meurs at al 1999 demonstrated the kinetics of MMPs and aggrecanases involvement in both early and late phase of the DMM-induced OA, using three other models of arthritis; reversible cartilage damage was induced in mice in the zymosan-induced arthritis (ZIA) model, partly irreversible cartilage damage in the antigen-induced arthritis (AIA) model, and irreversible, destructive cartilage damage in the collagen-induced arthritis (CIA) model^21^. Our data showed similar results where aggrecanase epitopes were induced before the appearance of VDIPEN epitopes, but they disappeared with progression of cartilage damage (compare our NVETEGE at 4 weeks vs 8 weeks). In contrast, VDIPEN epitopes in cartilage correlated with severe cartilage damage, but these epitopes were not detected during early PG degradation. This suggests a limited role for VDIPEN-inducing MMPs in early proteoglycan degradation during murine arthritis. Therefore, we suggest, based on our data, that protection of the proteoglycan would protect the collagen network. It is equally important to comment on the presence of NVTEGE staining in sham WT mice; Meurs *et al*. 1999 also showed that normal cartilage from young adult mice showed NVTEGE epitopes were already present to a limited extent before the disease onset^21^, which is also consistent with the demonstration of aggrecanase-induced neoepitopes in normal cartilage of either human^22^ or bovine origin^32^.

Furthermore, to understand why the overexpression of TIMP3 in mice did not similarly protect cartilage from degradation compared to [-1A]TIMP3-Tg mice, bone integrity must be investigated because increased levels of the inhibitor leads to skeletal abnormalities^27^. Indeed, one of the caveats of using a *Col2a1* or *Sox9* promoters to drive expression in cartilage is that these genes are also expressed in bone, as demonstrated in lineage tracing experiments during development^33^. Similarly, we previously reported that TIMP3 overexpressing mice homozygous for the transgene exhibit bone defects^27^. In this study, [-1A]TIMP3 mice do not show bone microarchitectural defects unlike TIMP3 mice, most likely because no bone defects seem to have been associated with the deficiency of aggrecanases during development. In addition, a recent study from Khokha’s lab^34^ used a genetic approach to test the contribution of aggrecan cleavage to the skeletal abnormalities seen in TIMP- deficient mice by crossing in Chloe or Jaffa knock-in mutations that block either MMP (Chloe) or ADAMTS (Jaffa) cleavage sites in the IGD of aggrecan^35,36^ showed that natural metalloprotease inhibitors are crucial regulators of chondrocyte maturation program, growth plate integrity, and skeletal proportionality.

In this study, the introduction of the TIMP3 variant,[-1A]TIMP3, that confers selective inhibition of aggrecanases provides a new therapeutic approach to treat OA. It also highlights the differences between classes of enzymes to target articular cartilage. While inhibiting aggrecanases maintains proteoglycan cleavage and protect collagen denaturation, there is an inverse relationship between type II collagen content and cleavage. While proteoglycan can be newly synthesised in OA cartilage, there is a lack of correlation between synthesis and the degradation of type II collagen which indicates that the latter aspects of turnover are not coordinated in the pathologic state;^37^ therefore, there is a need to maintain aggrecan integrity to minimize collagen type II degradation.

The most frequent side-effect observed in clinical trials of MMP inhibitors was the development of the MSS that manifested as pain and immobility in the shoulder joints, arthralgias, and contractures in the hands. An animal model of MSS has been previously established^38^ that specifically focuses on joint development and exhibits similarities in histopathology to those observed in human patients treated with MMP inhibitors.

Our study unequivocally shows that overexpression of [-1A]TIMP3, unlike TIMP3, does not have off-target effects that affect the skeletal formation and, in DMM model of OA it can protect articular cartilage from degradation by inhibiting aggrecanases. It remains to be investigated whether the degradation in TIMP3-Tg or [-1A]TIMP3-Tg mice can be stopped once the cartilage is already degraded, which has been demonstrated in MMP-13-knockout mice induced with OA^15^. For this, an inducible system that can express these transgenes in adult mice after the onset of injury at the desired time point will have to be generated in the future.

## Methods

### Generation of [-1A]TIMP3 transgenic mice

A bi-transgenic construct containing a collagen IIα1 chain (*Col2a1*) proximal promoter region (3000 bp), first exon (237 bp), and first intron (3020 bp) (gifted by B. de Crombrugghe)^39^ was used to induce the expression of human *[-1A]Timp3* or *Timp3* with a FLAG epitope tag (Asp-Tyr-Lys-Asp-Asp-Asp-Asp-Lys), internal ribosomal entry site (*IRES*) sequence, and *LacZ* with nuclear localizing signal. The transgene was microinjected into fertilized-C57BL/10 × CBA F1 eggs. Founder mice were identified by genomic DNA analysis using southern blot. [-1A]TIMP3 mRNA expression in the E15.5 embryo was confirmed by qRT-PCR using a human TIMP3-specific primer and probe (Qiagen). Transgenic animals, namely [-1A]TIMP3-Tg or TIMP3-Tg, were identified using a set of primers and TaqMan probe for β-galactosidase or 18S RNA as described previously^27^.

The creation of the transgenic mice and all studies were conducted under UK Home Office project licenses. Mice were housed in groups of up to 6 in individually ventilated cages maintained at 21 ± 2 °C on a 12-hour light/dark cycle with ad libitum food (RM3; Special Dietary Systems) and water. All experimental protocols were performed in compliance with the UK Animals (Scientific Procedures) Act 1986 regulations for the handling and use of laboratory animals. Mice were monitored daily for any health and welfare issues from birth, including any possible defects or significant change in size during the first two weeks. Mice were euthanised by an approved Schedule 1 method (by rising concentration of CO_2_)^27^. All experiments were approved by the ethics committee of the Kanazawa University Graduate School of Medical Science (IRB No. 352-2).

### Whole-mount X-gal staining

Whole-mount X-gal staining was performed as previously described^40^. Knee joints harvested from 2-week-old [-1A]TIMP3-Tg mice were fixed in 0.2% glutaraldehyde solution (0.1 M PBS pH 7.3, 5 mM EGTA, 2 mM MgCl_2_) for 15 min and stained with 1 mg/ml X-gal solution (0.1 M PBS pH 7.3, 2 mM MgCl_2_, 0.1% sodium deoxycholate, 0.2% NP-40, 5 mM potassium ferricyanide, and 5 mM potassium ferrocyanide) for up to 18 h at room temperature. For histological analysis, the samples were embedded in paraffin, and sagittal sections were generated and counterstained with Eosin.

### Measurement of β-galactosidase activity in embryo tissue homogenates

The Dual-Light System Kit (Applied Biosystems) was used to measure the β-galactosidase activity in E14.5 embryo tissue homogenate. Whole tail and limbs of E14.5 embryos (n = 5 forTIMP3-Tg heterozygotes and n = 7 for [-1A]TIMP3-Tg heterozygotes) were homogenized in lysis buffer using a micropestle (Eppendorf). The homogenate was centrifuged, and the clear supernatant obtained was subjected to analysis as per the manufacturer’s instructions. Light output was quantified as relative light units using a luminometer (Berthold Technologies). To normalize enzyme activities, the total protein concentration of lysates was determined using the BCA Protein Assay Kit (Pierce).

### Surgical induction of osteoarthritis

Ten-week-old mice were anesthetized using isoflurane, and microsurgery using a surgical microscope was performed using a previously described method to transect the meniscotibial ligament^14^, resulting in destabilization of the medial meniscus. The contralateral knee joint was sham-operated using the same approach without any ligament transaction. The animals were allowed to freely move with unrestricted access to food and water. Mice were sacrificed 4 and 8 weeks after surgery by CO_2_ inhalation. The sample number in each group was as follows: non-transgenic mice (WT), n = 40; TIMP3-Tg, n = 15; and [-1A]TIMP3-Tg, n = 24.

### Assessment of the progression and severity of osteoarthritis

Mice knee joints were fixed, decalcified, and embedded in paraffin, and frontal sections were taken through the entire joint. Slides were stained with Safranin-O and graded at 28-μm intervals through the joint by three scorers who were blinded to the specimen samples. The semiquantitative scoring system was modified from previous reports^41^. Each quadrant of the joint was graded between 0 (normal cartilage) and 6 (>80% loss of non-calcified cartilage). The scores were added from all levels to obtain the total histological score, which reflected the severity of osteoarthritis lesions as well as the surface area affected.

### Immunohistochemical analysis

Immunohistochemical localizations of FLAG and β-galactosidase were performed in paraffin sections. C-terminal aggrecan neoepitopes produced due to aggrecanase or MMP activity were assessed using anti-NVTEGE and anti-DIPEN antibodies (gifted by Dr John S Mort)^42,43^, respectively. To enhance the permeability of the extracellular matrix, glycosaminoglycans were removed by incubating the sections with protease-free chondroitinase ABC (Sigma). After blocking with 1% skimmed milk, sections were incubated with polyclonal rabbit antibodies against β-galactosidase (Abcam) and the NVTEGE epitope of aggrecan^43^ or with mouse monoclonal antibodies against the DIPEN epitope of aggrecan. Non-immune rabbit IgG or mouse IgG of the same dilution was used as the negative control instead of the primary antibodies. The sections were incubated with anti-rabbit IgG or anti-mouse IgG EnVision HRP enzyme conjugate (Dako), immersed in a diaminobenzidine solution to visualize any immunoreactivity, and then counterstained with hematoxylin.

### Micro-computed tomography (µCT)

Structural morphometric parameters were analyzed for tibial metaphyseal trabecular bone, cortical bone in diaphysis, and subchondral bone in epiphysis using micro-computed tomography (µCT) scans (Skyscan, Bruker, Belgium). Tibiae were isolated from 18-week old non-transgenic (n = 17), TIMP3-Tg heterozygote (n = 9), and [-1A]TIMP3-Tg heterozygote (n = 9) mice and were scanned with isotropic voxel size of 5 µm. Samples were reconstructed using NRecon v.1.4.4.0 and analyzed using CTAn v.1.5.1.3. software.

### Statistical analysis

For comparisons between three groups, data were analyzed by one-way ANOVA and Dunnett’s test using SPSS, version 23 (IBM) or t-test for comparisons between two groups. A *P*-value of less than 0.05 was considered to indicate a statistically significant difference.

## Supplementary information

Supplementary information.
