# Supplementary material for: Aggrecanase-selective tissue inhibitor of metalloproteinase-3 (TIMP3) protects articular cartilage in a surgical mouse model of osteoarthritis
Source: Sci Rep. 2020 Jun 9;10:9288. doi: 10.1038/s41598-020-66233-0 (PMC7283274; doi:10.1038/s41598-020-66233-0)
Supplement: Supplementary file 1 — Supplementary information. [file 41598_2020_66233_MOESM1_ESM.pdf]

Supplementary Information

Aggrecanase-selective tissue inhibitor of metalloproteinase-3 (TIMP3) protects articular cartilage in a surgical mouse model of osteoarthritis

Hiroyuki Nakamura[1, 2*], Phoung Vo[2], Ioannis Kanakis[3], Ke Liu[3], and George Bou-Gharios[3]

[1]Department of Oral and Maxillofacial Surgery, Kanazawa University Graduate School of Medical Science Kanazawa, Ishikawa, Japan. [2]Matrix Biology Department, the Kennedy Institute of Rheumatology Division, Imperial College London, Hammersmith, London, UK. [3]Institute of Ageing and Chronic Disease, University of Liverpool, William Henry Duncan Building, Liverpool, UK
*e-mail: hnak@me.com

Supplementary Figure S1: Representative images of Safranin-O stained sections of the medial condyle and tibial plateau of non-transgenic mice (WT), TIMP3-Tg heterozygous, and [-1A] TIMP3-Tg heterozygous in skeletally mature mice at 18 weeks of age to show similarity of the articular cartilage proteoglycan composition. Bars, 200 µm.

Supplementary Figure S2: (a) Comparison of expression in transgenic mice by determining β-galactosidase activity in [-1A]TIMP3 line 7 (n=7) and [-1A]TIMP3 line (n=5, line 7). Values represent the mean ± SEM. (b) Representative images of Safranin-O stained sections from [-1A]TIMP3 lines 7 and 13 showing that cartilage was protected in mice expressing high levels of the [-1A]TIMP3 transgene (line 7) but not in low expressing transgenic line 13. (c) Histological maximum and summed scores of joints, 8 weeks after the induction of DMM or sham operated control

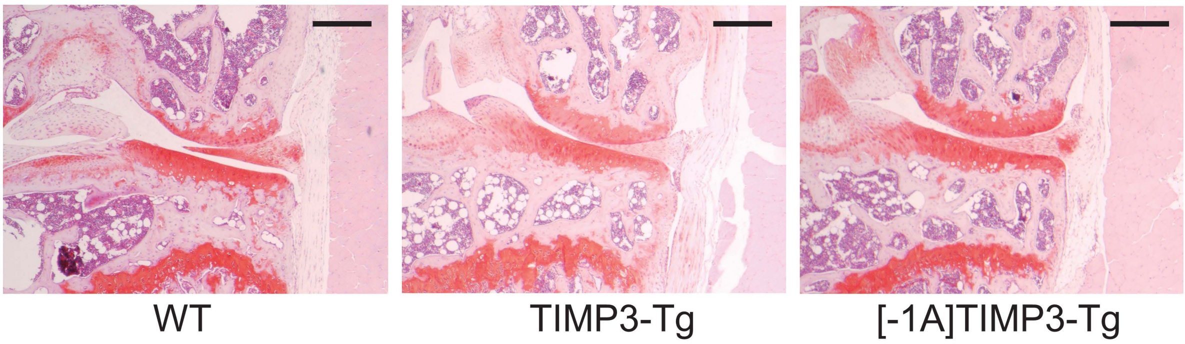
WT
TIMP3-Tg
[-1A]TIMP3-Tg

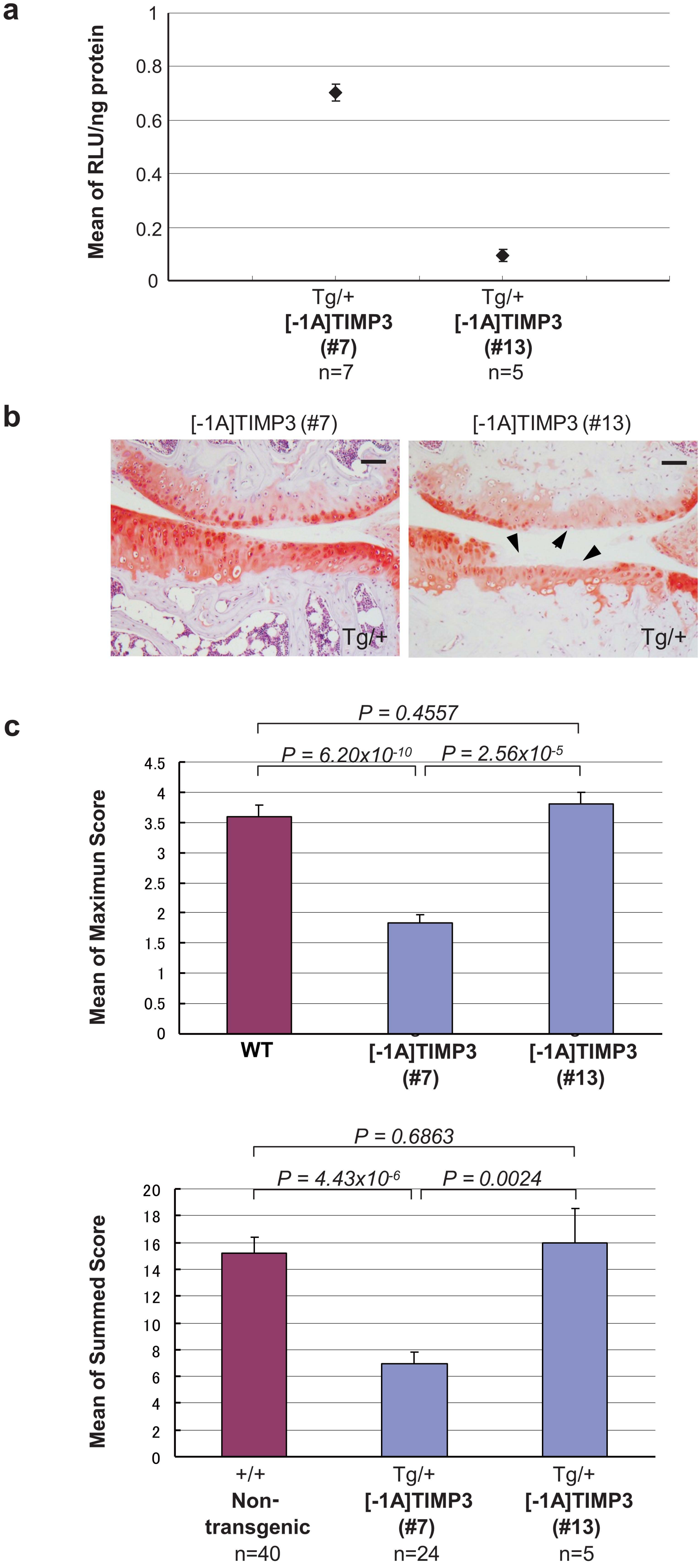
a
Mean of RLU/ng protein
1
0.8
0.6
0.4
0.2
0
Tg/+
[-1A]TIMP3
(#7)
n=7
Tg/+
[-1A]TIMP3
(#13)
n=5
b
[-1A]TIMP3 (#7)
[-1A]TIMP3 (#13)
Tg/+
Tg/+
c
P = 0.4557
P = 6.20x10-10
P = 2.56x10-5
Mean of Maximun Score
4.5
4
3.5
3
2.5
2
1.5
1
0.5
0
WT
[-1A]TIMP3
(#7)
[-1A]TIMP3
(#13)
P = 0.6863
P = 4.43x10-6
P = 0.0024
Mean of Summed Score
20
18
16
14
12
10
8
6
4
2
0
+/+
Non-
transgenic
n=40
Tg/+
[-1A]TIMP3
(#7)
n=24
Tg/+
[-1A]TIMP3
(#13)
n=5
